# Wandering Spleen: A Rare Case of an Adnexal Lesion

**DOI:** 10.7759/cureus.18231

**Published:** 2021-09-23

**Authors:** Panagiotis Vlastarakos, Angeliki Rouvali, Maria Giourga, Angeliki Gerede, Ekaterini Domali

**Affiliations:** 1 1st Department of Obstetrics and Gynecology, General Hospital of Athens ‘Alexandra Hospital’, National and Kapodistrian University of Athens School of Medicine, Athens, GRC; 2 Department of Obstetrics and Gynecology, Democritus University of Thrace Alexandroupolis, Greece, Alexandroupolis, GRC

**Keywords:** lesion, adnexal, ultrasound, spleen, wandering

## Abstract

The wandering spleen (WS) is a rare condition in which the spleen is not found in its usual location in the left hypochondrium but is positioned in the lower abdomen or the pelvis. This is a case of a 21-year-old woman who presented with chronic, intermittent, and subtle pain in the left lower quadrant of her abdomen. After clinical examination and ultrasound evaluation, an adnexal lesion was detected in the left lumbar area, and no splenic tissue was found in the left hypochondrium. The wandering spleen should be included in the differential diagnosis when encountering a patient with non-typical or acute abdominal pain. Accurate diagnostic evaluation can be performed with low-cost imaging modalities such as Doppler ultrasound.

## Introduction

The wandering spleen (WS) is a medical condition in which the spleen is absent from its customary placement in the left hypochondrium but is positioned in the lower abdomen or the pelvis [1]. The reason for this translocation is the laxity or the absence of peritoneal attachments, including the lienorenal and gastrosplenic ligaments. Although splenomegaly, pregnancy, and injury can be the cause of this laxity, many cases are congenital. The majority of the patients with wandering spleen manifest no symptoms; thus, the diagnosis is usually established only by routine abdominal examination or at a hospital emergency department when the patient is seeking care due to acute abdominal pain, vomiting, or obstipation [2].

Adnexal lesions represent a frequent condition across women of any age. Ultrasound is the first-line imaging modality for the evaluation of suspected adnexal masses [3]. According to the International Ovarian Tumor Analysis (IOTA) group, an adnexal lesion is defined as "the portion of an ovary or adnexal mass that is assessed to be incompatible with normal physiologic function based on an assessment of ultrasound scans" [4]. We present a case of a young woman complaining of chronic, subtle, remittent pain in the left lumbar region with a smooth, solid lesion detected on transvaginal ultrasonography.

## Case presentation

On February 8, 2020, a 21-year-old woman who originated from Syria presented to our Gynecological Ultrasonography Unit's outpatient department on a scheduled appointment. She was a mother of one child, delivered vaginally four years ago, and breastfed until the age of 12 months. She was a non-smoker, her body mass index (BMI) was 23.6 kg/m^2^, and her menstrual period was regular every month. Her personal medical history was uneventful (including drug, family, and psychosocial history) since she was under no medical treatment and had been neither hospitalized nor operated on in the past. The patient complained of chronic, intermittent, and subtle pain in the left lower quadrant of her abdomen in the past four years, occurring randomly throughout the year and commencing after the birth of her child.

**Figure 1:**
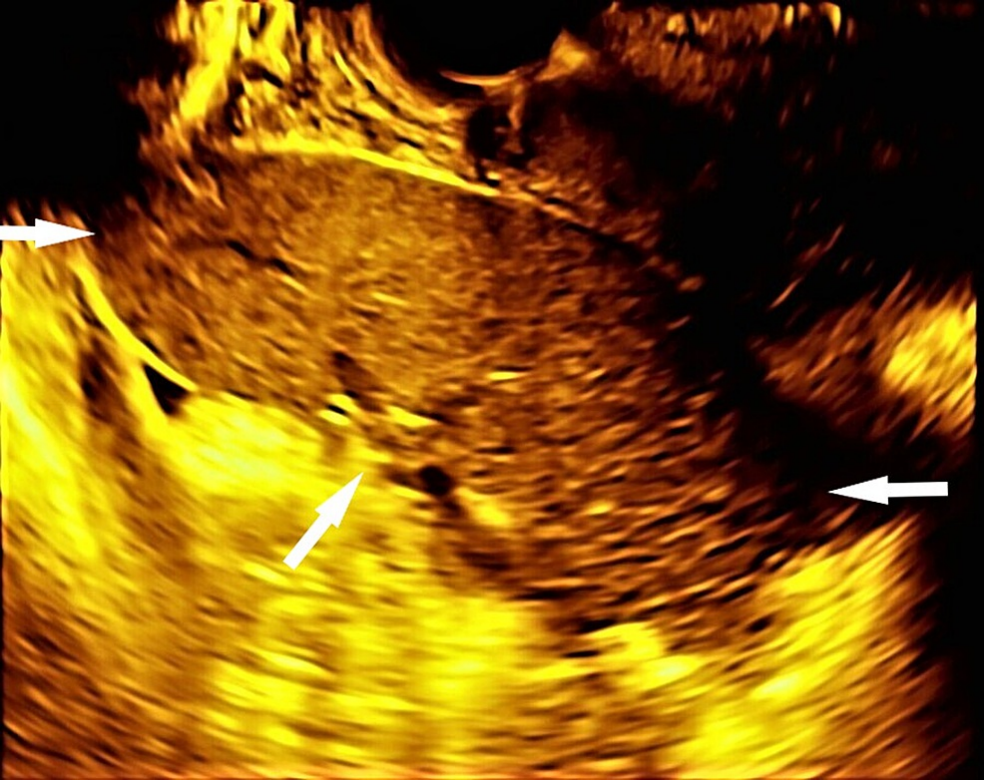
Two-dimensional grayscale transvaginal ultrasonography revealed a comma-shaped lesion (white arrows) in the left lumbar region

Upon arrival, bilateral pelvic examination revealed a mobile anteverted uterus of average size with no cervical tenderness during the translocation of the cervix. The palpation of the right fornix was unremarkable, whereas, at the left fornix, a palpable, smooth mobile mass was noticed. On transvaginal ultrasound (TVUS), the imaging of the uterus and the adnexa were normal. However, a solid, smooth, comma-shaped lesion measuring 11 * 4.5 * 11.5 centimeters was discovered at the left lumbar region, clearly delimited from the left ovary (Figure 1).

**Figure 2:**
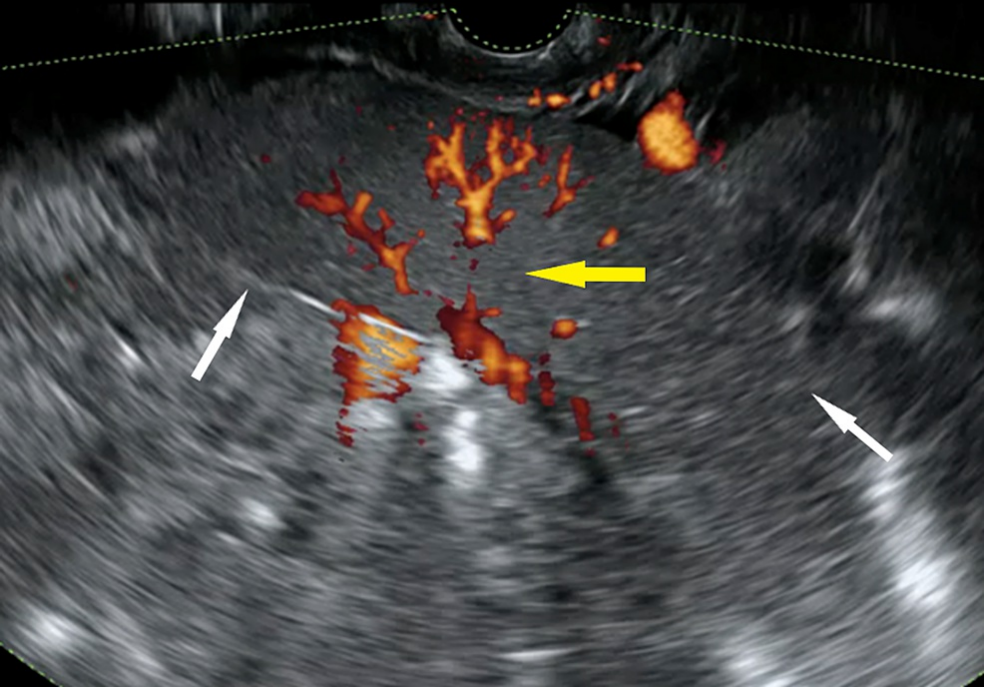
Doppler-enhanced transvaginal ultrasonography revealed central vascularity (yellow arrow) of the lesion (white arrows)

Power Doppler-enhanced transvaginal ultrasonography (TVUS) demonstrated central vascularity of the lesion (Figure 2).

As the next step of the diagnostic procedure, transabdominal ultrasound (TAUS) was performed, which revealed no splenic tissue in the left hypochondrium, with no other findings. Routine biochemical parameters were in the normal range except for mild leukopenia (3400/mm^3^), whereas hematologic and microbiologic investigations were all found as expected.

Given the clinical examination findings and ultrasound evaluation, the diagnosis of ectopic spleen was established, and the patient was advised to report to the outpatient department of the surgical clinic for further management and treatment.

## Discussion

The wandering spleen is an infrequent clinical entity that occurs in less than 0.5% of splenectomies and is defined by the spleen being positioned ectopically within the abdomen or pelvis due to its prolonged vascular pedicle [5]. A protracted splenic mesentery and increased splenic mobility result from the complete lack or elongation of the splenic suspensory ligaments [6]. The etiological factors include congenital omission of the ligaments that retain the spleen in its normal anatomic position or slackening of those ligaments resulting from conditions like trauma and abdominal surgery or pregnancy, as in our patient [7]. In the scenario of agenesis, the dorsal mesogastrium fails to fuse with the posterior abdominal wall during embryogenesis [6].

The wandering spleen can be diagnosed at any age. The age distribution ranges from newborn to 81 years of age with two separate distribution peaks. One-third of the patients are children, predominantly in the first year of life [8]. However, it occurs more frequently in women of reproductive age, particularly in pregnant women. Weakening of the abdominal wall and hormonal changes during pregnancy are thought to be the etiological factors for ligamentous strengthening and the higher prevalence of this uncommon ailment in pregnant and postpartum females [9].

The condition can be asymptomatic or emerge as acute abdominal pain due to splenic torsion with secondary infarction, in terms of clinical manifestation. However, most frequently, patients are referred to a physician due to subtle, nonspecific, chronic abdominal pain. A palpable mass may be identified during a clinical examination. The nonspecific clinical symptoms make this diagnosis extremely challenging to make clinically [2]. Noninvasive imaging modalities are playing a crucial role in the diagnosis of this sparse medical entity. Pelvic and abdominal ultrasonography enhanced by power Doppler, magnetic resonance imaging (MRI), computed tomography (CT), and angiography can provide valuable information and lead to a specific diagnosis [10]. Abdominal ultrasonography may reveal a comma-shaped spleen in an ectopic location and an absence of splenic tissue in the left hypochondrium [11]. Doppler sonography can accurately estimate blood flow in the splenic vasculature and confirm the suspected torsion. However, a multi-slice spiral CT scan is considered the gold standard in order to determine the location and viability of the spleen [12].

The treatment of a wandering spleen is surgical. Whereas splenectomy was considered the gold standard until recently, splenopexy seems to be more preferred since awareness of the crucial splenic function and the concern regarding the overwhelming post-splenectomy infection (OPSI) are increasing [13]. Spleen preservation is strongly recommended in clinical practice. In the absence of an indication of splenic infarction, de-torsion of the splenic pedicle and splenopexy are suitable surgical options, with the laparoscopic approach being encouraged [14].

## Conclusions

Wandering spleen is an uncommon medical condition with possible life-threatening consequences. Therefore, clinicians and radiologists must be concerned regarding this possible diagnosis when encountering a patient with non-typical or early-onset severe abdominal pain. Accurate diagnostic evaluation can be achieved with low-cost imaging modalities, such as Doppler ultrasound, and every effort should prioritize the preservation of the spleen. Splenopexy is a reasonable procedure that is minimally invasive and bears no impact on splenic function, whereas splenectomy is usually recommended once splenic necrosis is present.
